# Influence of source–detector separation on diffuse correlation spectroscopy measurements of cerebral blood flow with a multilayered analytical model

**DOI:** 10.1117/1.NPh.9.3.035002

**Published:** 2022-07-20

**Authors:** Hongting Zhao, Erin M. Buckley

**Affiliations:** a Wallace H. Coulter Department of Biomedical Engineering at Georgia Institute of Technology and Emory University, Atlanta, Georgia, United States; b Emory University School of Medicine, Department of Pediatrics, Atlanta, Georgia, United States; c Children’s Healthcare of Atlanta, Children’s Research Scholar, Atlanta, Georgia, United States

**Keywords:** diffuse correlation spectroscopy, cerebral blood flow

## Abstract

**Significance:**

Diffuse correlation spectroscopy (DCS) is an emerging noninvasive optical technology for bedside monitoring of cerebral blood flow. However, extracerebral hemodynamics can significantly influence DCS estimations of cerebral perfusion. Advanced analytical models can be used to remove the contribution of extracerebral hemodynamics; however, these models are highly sensitive to measurement noise. There is a need for an empirical determination of the optimal source–detector separation(s) (SDS) that improves the accuracy and reduces sensitivity to noise in the estimation of cerebral blood flow with these models.

**Aim:**

To determine the influence of SDS on solution uniqueness, measurement accuracy, and sensitivity to inaccuracies in model parameters when using the three-layer model to estimate cerebral blood flow with DCS.

**Approach:**

We performed a series of *in silico* simulations on samples spanning a wide range of physiologically-relevant layer optical properties, thicknesses, and flow. Data were simulated at SDS ranging from 0.5 to 3.0 cm using the three-layer solution to the correlation diffusion equation (with and without noise added) and using three-layer slab Monte Carlo simulations. We quantified the influence of SDS on uniqueness, accuracy, and sensitivity to inaccuracies in model parameters using the three-layer inverse model.

**Results:**

Two SDS are required to ensure a unique solution of cerebral blood flow index (CBFi). Combinations of 0.5/1.0/1.5 and 2.5 cm provide the optimal choice for balancing the depth penetration with signal-to-noise ratio to minimize the error in CBFi across a wide range of samples with varying optical properties, thicknesses, and dynamics.

**Conclusions:**

These results suggest that the choice of SDS is critical for minimizing the estimated error of cerebral blood flow when using the three-layer model to analyze DCS data.

## 1 Introduction

 Diffuse correlation spectroscopy (DCS) is an increasingly popular noninvasive technology that uses near-infrared light for portable, bedside monitoring of cerebral blood flow.^1^^,^^2^ By the nature of the measurement, the light must travel through the scalp and skull to reach the brain and return to the tissue surface. Thus, extracerebral hemodynamics can significantly influence DCS estimations of cerebral perfusion.^2^ To minimize these extracerebral contributions, several approaches have been proposed that typically fall into one of two categories: (1) hardware modifications or (2) improved analytical modeling. On the hardware side, developments include methods that enhance depth sensitivity, either through time domain^3^^,^^4^ or interferometric approaches,^5^^,^^6^ or by moving to the second optical window at 1064 nm.^7^ While these approaches are exciting and will likely be the future of DCS, limitations related to detector speed, availability, and cost currently limit widespread adoption. Alternatively, improved analytical modeling techniques that aim to remove the contribution of extracerebral hemodynamics by modeling the head as a layered medium and isolating the signal arising from the brain layer are also available. Although numerous studies have investigated a two-layer model that consists of an extracerebral layer containing scalp, skull, and cerebrospinal fluid (CSF) along with a cerebral layer containing gray and white matter,^8^^–^^11^ a more complex three-layer model (scalp, skull, and brain) allows for consideration of the negligible blood flow in the skull.^12^^–^^14^ Recent validation with transcranial Doppler ultrasound (TCD) supports the superiority of the three-layer models over two-layer models and highlights the need for including a negligible flow layer (skull) when assessing cerebral perfusion with DCS.^15^

Although the importance and accuracy of the three-layer slab analytical model have been investigated in former studies,^12^^,^^14^^,^^16^ the approach is highly sensitive to measurement noise. Thus, it is important to balance the depth penetration with the signal-to-noise ratio (SNR) when selecting source–detector separation(s) (SDS) to optimize the accuracy of cerebral blood flow estimation using this approach. To date, studies that utilize the three-layer model have selected SDS(s) somewhat subjectively.^12^^–^^15^ There is a need for an empirical determination of the optimal SDS that improves the accuracy and reduces the sensitivity to noise of the estimation of cerebral blood flow with the three-layer model.

In this study, we use a series of *in silico* experiments to determine the influence of SDS on solution uniqueness, measurement accuracy, and sensitivity to inaccuracies in model parameters when using the three-layer model. Our results can be used to guide the design of future experiments using the three-layer model to estimate cerebral blood flow with traditional continuous-wave DCS systems.

## 2 Methods

**Fig. 1:**
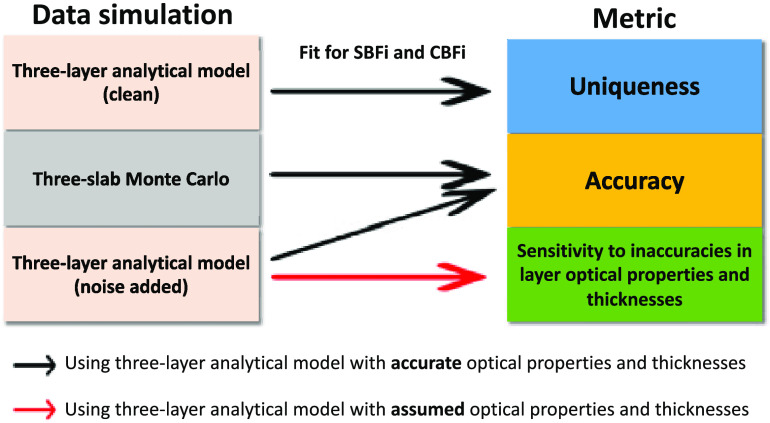
Scheme of the experimental design.

To determine the influence of the choice of SDS on uniqueness, accuracy, and sensitivity to inaccuracies in model parameters, we simulated data across a wide range of physiologically-relevant layer optical properties, thicknesses, and flow indices. Data were simulated at SDSs ranging from 0.5 to 3.0 cm using the three-layer solution to the correlation diffusion equation (with and without noise added) and using three-layer slab Monte Carlo (MC) simulations. Next, simulated data were fit to the three-layer analytical model for scalp and cerebral blood flow indices using either known (true) or assumed layer optical properties and thicknesses. Finally, the error in the estimated cerebral blood flow index was calculated. In the following sections, we detail each of these steps, and we outline the approach in Fig. 1.

### 2.1 Three-Layer Analytical Model Simulations

Data were simulated using the three-layer solution to the correlation diffusion equation for the normalized intensity autocorrelation function, $g_{2}(r,\tau )$, at delay time, τ, and SDS, r, as in Refs. 12 and 16. To investigate the influence of r, data were simulated at 0.5, 1.0, 1.5, 2.0, 2.5, and 3 cm. For each r, we simulated $g_{2}(r,\tau )$ at a range of optical properties and skull/scalp thicknesses ($\mu_{a,\text{scalp}}\in [0.05,0.15]\;{\mathrm{cm}}^{-1}$, $\mu_{a,\text{skull}}\in [0.05,0.15]\;{\mathrm{cm}}^{-1}$, $\mu_{a,\text{brain}}\in [0.05,0.25]\;{\mathrm{cm}}^{-1}$, $\mu_{s,\text{scalp}}^{\prime }\in [8,12]\;{\mathrm{cm}}^{-1}$, $\mu_{s,\text{skull}}^{\prime }\in [8,12]\;{\mathrm{cm}}^{-1}$, $\mu_{s,\text{brain}}^{\prime }\in [2,6]\;{\mathrm{cm}}^{-1}$, $L_{\text{scalp}}\in [0.15,0.53]\;\mathrm{cm}$, and $L_{\text{skull}}\in [0.35,1.10]\;\mathrm{cm}$).^16^ For each parameter, we sampled 11 evenly spaced values within the reported range while fixing the other parameters at the median of the corresponding range. For each of these 11 values, we simulated 12 evenly spaced values of CBFi $\in [2\times 10^{-8},9\times 10^{-8}]\;{\mathrm{cm}}^{2}/s$ and six values of scalp blood flow index (SBFi) ∈[1/8,1/3]×CBFi. Thus, in total, we simulated 38,016 clean (no noise added) $g_{2}(r,\tau )$ curves (8 parameters × 11 values/parameter × 12 CBFi × 6 SBFi × 6 separations = 38,016).

Next, noise was added to simulated $g_{2}(r,\tau )$ curves using the following formula for the standard deviation [σ(τ)] of the $g_{2}(r,\tau )$ model:^17^

$$\sigma (\tau )=\sqrt{T/t}[\beta^{2}\frac{(1+e^{-2\Gamma T})(1+e^{-2\Gamma \tau })+2m(1-e^{-2\Gamma T})e^{-2\Gamma \tau }}{1-e^{-2\Gamma T}}\;{+2{\left\langle n\right\rangle }^{-1}\beta (1+e^{-2\Gamma \tau })+{\left\langle n\right\rangle }^{-2}(1+\beta e^{-\Gamma \tau })]}^{-1/2},$$

where Γ is the decay rate of $g_{2}(r,\tau )$, T is the bin width of the correlator, m is the bin index corresponding to decay time τ, ⟨n⟩ is the average number of photons detected within bin time T, t is the total averaging time, and β is a coherence factor. To mimic experimental conditions, we simulated a DCS system with eight total detectors. Often, multiple detector fibers are bundled together at a single SDS to increase the SNR. Thus, we investigated two situations, one wherein all eight detectors are positioned at the same SDS, and another wherein one detector is positioned at a small SDS (SDS1) and seven detectors are positioned at a large SDS (SDS2). For the first situation, we simulated a single SDS of 0.5, 1.0, 1.5, 2.0, 2.5, or 3 cm. For the second situation, we simulated 30 combinations of SDS1 and SDS2, wherein SDS1 ranged from 0.5 to 2.5 cm (step size 0.5 cm) and SDS2 ranged from 1 to 3 cm (step size 0.5 cm). To further mimic typical experimental conditions, we used T and τ settings of the Flex08OEM hardware correlator (Flex05-8ch, Correlator.com, New Jersey, United States) and assumed a detected intensity, I≡⟨n⟩/T, at 2.5 cm of 20 kHz. Intensities at all other separations were scaled accordingly for each sample using the normalized field correlation function at delay time zero, $G_{1}(0)$,^1^^,^^12^ with the known layer thicknesses and optical properties of the sample. Three different noise levels were simulated by employing averaging times, t, of 1, 3, 10, or 30 s.

### 2.2 Three-Layer Slab Monte Carlo Simulations

Voxel-based MC simulations were performed on a $200\times 200\times 200\;{\mathrm{mm}}^{3}$ sample volume segmented into three layers (scalp, skull, and brain). Due to limited computational resources, a range of layer thicknesses and optical properties were not simulated as in our analytical simulations. Instead, we simulated a single setup for layer thickness and optical properties wherein the scalp layer was 3-mm thick, the skull layer was 7-mm thick,^18^^,^^19^ and the optical properties of each layer were set to the median of the simulated ranges in Sec. 2.1. The anisotropic factor (g) and index of refraction (n) of each layer were fixed at 0.89^20^ and 1.4,^21^ respectively. Detectors (1 mm in diameter) were spaced 0.5 to 3.0 cm from the source with a step size of 0.5 cm. Simulations were performed with MC eXtreme (MCX).^20^ Because MCX limits the total number of detected photons per simulation to 1 million, simulations were run separately for each SDS to ensure that a sufficient number of photons were detected.

For each detected photon, MCX records the momentum transfer, scattering angle, and total pathlength traveled in each layer. This information was used to calculate the unnormalized electric autocorrelation function $G_{1}(r,\tau )$^22^

$$G_{1}(r,\tau )=\frac{1}{N_{p}}\overset{N_{p}}{\underset{n=1}{\sum }}\;\exp(\overset{N_{\text{tis}}}{\underset{i=1}{\sum }}-\frac{1}{3}Y_{n,i}k_{0}^{2}{\left\langle \Delta r^{2}(\tau )\right\rangle }_{i})\exp(-\overset{N_{\text{tis}}}{\underset{i=1}{\sum }}\mu_{a,i}L_{n,i}).$$ (1)

Here $N_{p}$ is the number of detected photons at separation r, $N_{\mathrm{tis}}$ is the number of tissue types (three for our simulations), $Y_{n,i}$ is the dimensionless momentum transfer for the n’th photon in the i’th tissue type, $L_{n,i}$ is the total path length of the n’th photon in the i’th tissue type, and $\mu_{a,i}$ is the absorption coefficient of the i’th tissue type. As in Sec. 2.1, we assumed that the mean square displacement of the i’th layer (${\left\langle \Delta r^{2}(\tau )\right\rangle }_{i}$) took the form of $6D_{i}\tau$, where $D_{i}$ is the effective diffusion coefficient of the i’th layer. We simulated 12 values for CBFi (i.e., $D_{3}$) $\in [2\times 10^{-8},9\times 10^{-8}]\;{\mathrm{cm}}^{2}/s$ and six values of SBFi (i.e., $D_{1}$) ∈[1/8,1/3]×CBFi. We assumed that blood flow in the skull is negligible, i.e., $D_{2}=0$.^15^ Thus, in total, we simulated 432 $G_{1}(\tau )$ curves (12 CBFi × 6 SBFi × 6 separations = 432). Each simulated $G_{1}(r,\tau )$ was normalized to $G_{1}(r,0)$, and then the Siegert relationship with β=0.5 was used to estimate $g_{2}(r,\tau )$.^1^

### 2.3 Cerebral Blood Flow Index Estimation

First, to investigate how the choice of SDS influences the uniqueness of the CBFi estimation, simulated clean $g_{2}(r,\tau )$ from the analytical model at either one or two SDS were simultaneously fit for CBFi and SBFi using the three-layer solution to the correlation diffusion equation. Data were fit using a single cost function

$$\chi^{2}=\overset{N_{r}}{\underset{j=1}{\sum }}\overset{N_{\tau }}{\underset{k=1}{\sum }}{\left[{g_{2}}_{\text{simulated}}(r_{j},\tau_{k},{\mathrm{CBFi}}_{\text{true}},{\mathrm{SBFi}}_{\text{true}})-g_{2}(r_{j},\tau_{k},{\mathrm{CBFi}}_{\text{estimated}},{\mathrm{SBFi}}_{\text{estimated}})\right]}^{2},$$

where $N_{r}$ is the number of SDSs and $N_{\tau }$ is the number of τ. We minimized $\chi^{2}$ using *fminsearchbnd*^23^ in MATLAB^®^ (Mathworks). Fitting bounds for both CBFi and SBFi were set to $[1\times 10^{-11},1\times 10^{-6}]\;{\mathrm{cm}}^{2}/s$. For these fits, it was assumed that the optical properties and layer thickness were known. The percentage error in CBFi was defined as $({\mathrm{CBFi}}_{\text{estimated}}-{\mathrm{CBFi}}_{\text{true}})/{\mathrm{CBFi}}_{\text{true}}\times 100\%$. Nonunique solutions were identified as those in which the error in CBFi estimation was nonzero.

Next, to assess how the accuracy of the three-layer model depends on the choice of SDS, we compared the accuracy of CBFi estimated from simulated, noise-added $g_{2}(r,\tau )$ with the analytical model and from simulated MC $g_{2}(r,\tau )$ that were simultaneously fit for CBFi and SBFi as described in the previous paragraph.

Finally, to assess sensitivity of estimation of CBFi to inaccuracies in assumed model parameters at a different choice of SDSs, we fit simulated noise-added data with assumed optical properties and thicknesses, which are off from true values. Assumed values for each parameter $\left(\mu_{a,n},\mu_{s,n}^{\prime },L_{n}\right)$ were fixed as the median of the range in Sec. 2.1. Then, we calculated the mean and standard deviation of error in estimated CBFi at different inaccuracies in layer optical properties and thicknesses. A bigger mean/standard deviation error means higher sensitivity of estimation to inaccuracies in prior information.^16^

## 3 Results

### 3.1 Uniqueness of the Three-Layer Inverse Model Depends on Number of Source–Detector Separations

**Fig. 2:**
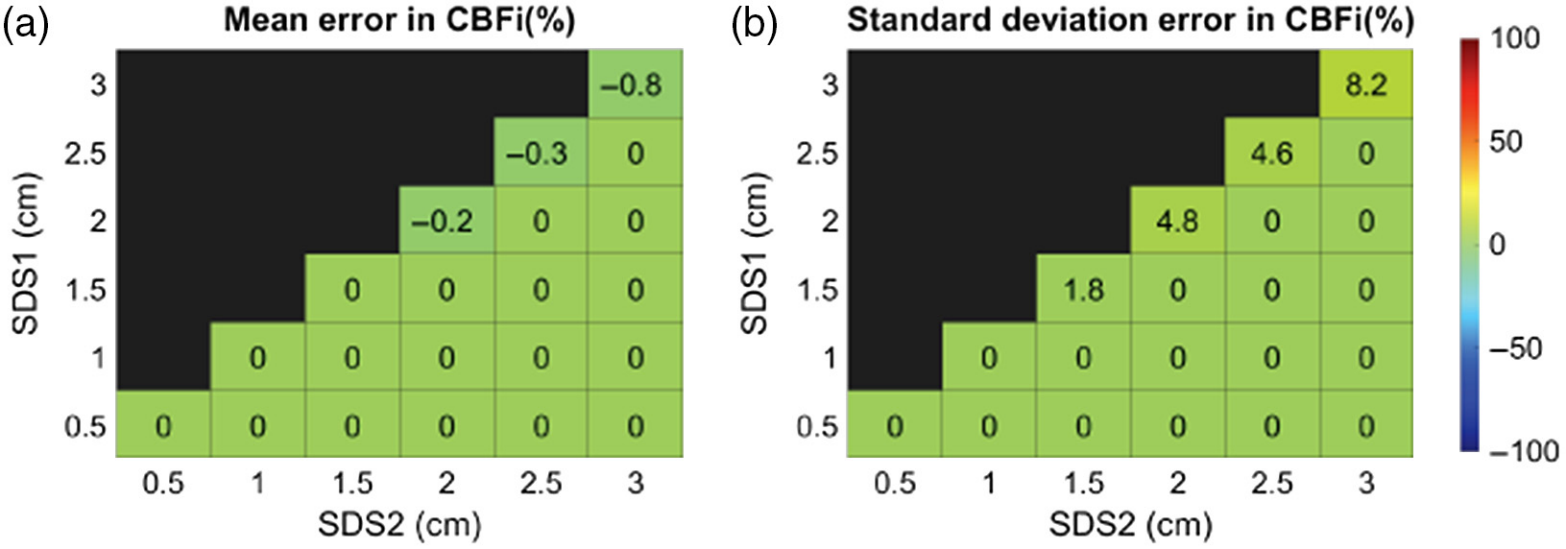
Error of estimated CBFi using different SDS. Each box represents the (a) mean and (b) standard deviation in the error of CBFi across all samples simulated with the three-layer analytical model (no noise added) with varying layer optical properties, thicknesses, and scalp and cerebral blood flow indices (6336 total) for the SDS combination given by the row and column.

**Fig. 3:**
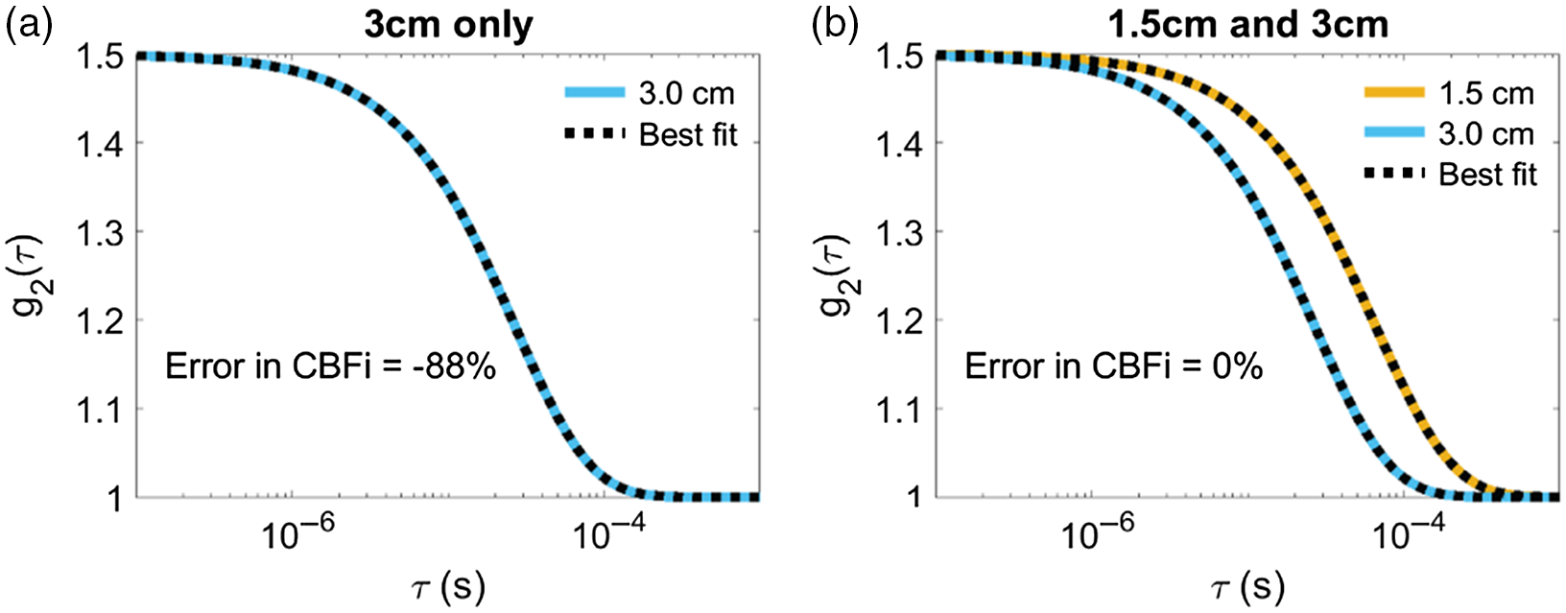
Representative $g_{2}(\tau )$ data simulated from a sample with $\mathrm{SBFi}=6.7\times 10^{-9}$ and $\mathrm{CBFi}=2.0\times 10^{-8}\;{\mathrm{cm}}^{2}/s$. (a) When fitting only data from 3.0 cm, the estimated value of $\mathrm{SBFi}=2.1\times 10^{-8}$ and $\mathrm{CBFi}=2.5\times 10^{-9}\;{\mathrm{cm}}^{2}/s$, resulting in an error in estimated CBFi of 88%. (b) When fitting data from 1.5 and 3.0 cm simultaneously, the estimated value of $\mathrm{SBFi}=6.7\times 10^{-9}\;{\mathrm{cm}}^{2}/s$ and $\mathrm{CBFi}=2.0\times 10^{-8}\;{\mathrm{cm}}^{2}/s$, resulting in an error in estimated CBFi of 0%. In each subplot, the orange line is the simulated curve at 1.5 cm, the blue line is the simulated curve at 3.0 cm, and the black dashed lines denoted the best fit to the data. The optical properties of this sample were $\mu_{a,\text{scalp}}=0.1\;{\mathrm{cm}}^{-1}$, $\mu_{a,\text{skull}}=0.1\;{\mathrm{cm}}^{-1}$, $\mu_{a,\text{brain}}=0.15\;{\mathrm{cm}}^{-1}$, $\mu_{s,\text{scalp}}^{\prime }=10\;{\mathrm{cm}}^{-1}$, $\mu_{s,\text{skull}}^{\prime }=10\;{\mathrm{cm}}^{-1}$, and $\mu_{s,\text{brain}}^{\prime }=4\;{\mathrm{cm}}^{-1}$. Scalp and skull thickness were 0.35 and 0.73 cm, respectively.

When using the three-layer inverse model on simulated, no-noise-added three-layer data from a single SDS, we found a handful of samples wherein a unique solution does not exist for SDS≥1.5  cm (as indicated by nonzero mean/stdev error in CBFi in the diagonal of Fig. 2). However, when using two SDS, the error in estimated CBFi across all samples was zero (Fig. 2). To illustrate this point, Fig. 3(a) shows a representative curve at SDS=3  cm for which a unique solution was not observed. Although the fitted curve matches the simulated data well, the estimated CBFi is off from the true value by 88%. For this sample, by adding a second SDS at 1.5 cm to the inverse problem, CBFi was recovered accurately [Fig. 3(b)]. We note that interpretation of Fig. 3 must be met with caution. Due to the inverse crime,^24^ an error of 0 in Fig. 3 does not imply that a unique solution exists; however, a nonzero error confirms the lack of a unique solution.

### 3.2 Accuracy of Three-Layer Inverse Model Depends on the Choice of Source–Detector Separations

**Fig. 4:**
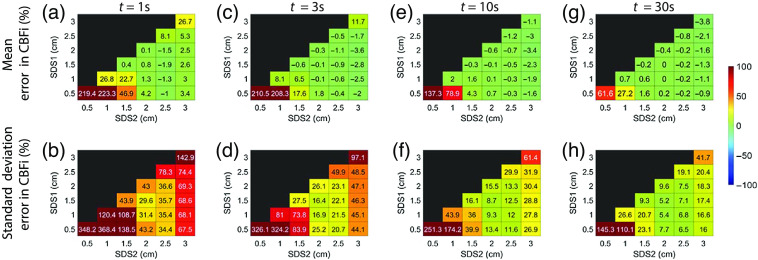
(a), (c), (e), and (g) Mean and (b), (d), (f), and (h) standard deviation of the percentage error in estimated CBFi at different integration times [(a) and (b) t=1  s; (c) and (d) 3 s; (e) and (f) 10 s; (g) and (h) 30 s] across all simulated samples (6336 total) of varying layer optical properties, thicknesses, and scalp and cerebral blood flow indices for the SDS combination given by the row and column.

Using noise-added three-layer data, we found that the average error in CBFi across all samples tested is small when a single SDS is used to estimate CBFi (diagonals of each subplot in Fig. 4); however, the standard deviation in this error is appreciable because there are some samples that are highly sensitive to noise and/or that lack a unique solution. Error is highest when using a single small SDS (0.5 or 1 cm), presumably due to poor brain sensitivity, as well as when using a large SDS (3 cm), presumably due to a poor SNR.

Compared with a single SDS, two SDS yield a smaller mean/stdev error in CBFi across all samples for the majority of SDS combinations tested in Fig. 4. The error is smallest when at least one of the two SDS is at 2.0 or 2.5 cm. In contrast, the error is larger when one of the two SDS is at 3.0 cm (as indicated by the large standard deviation for these SDS combinations) due to the low SNR at this separation.

In general, the three-layer model is highly susceptible to the low SNR. Thus, while the trends in CBFi accuracy as a function of SDS persisted for all noise levels, longer averaging times yielded greater accuracy, as indicated by the reduction in the standard deviation in the error in CBFi across all samples tested with increasing the integration time (i.e., with increasing the SNR), shown in the bottom row of Fig. 4.

**Fig. 5:**
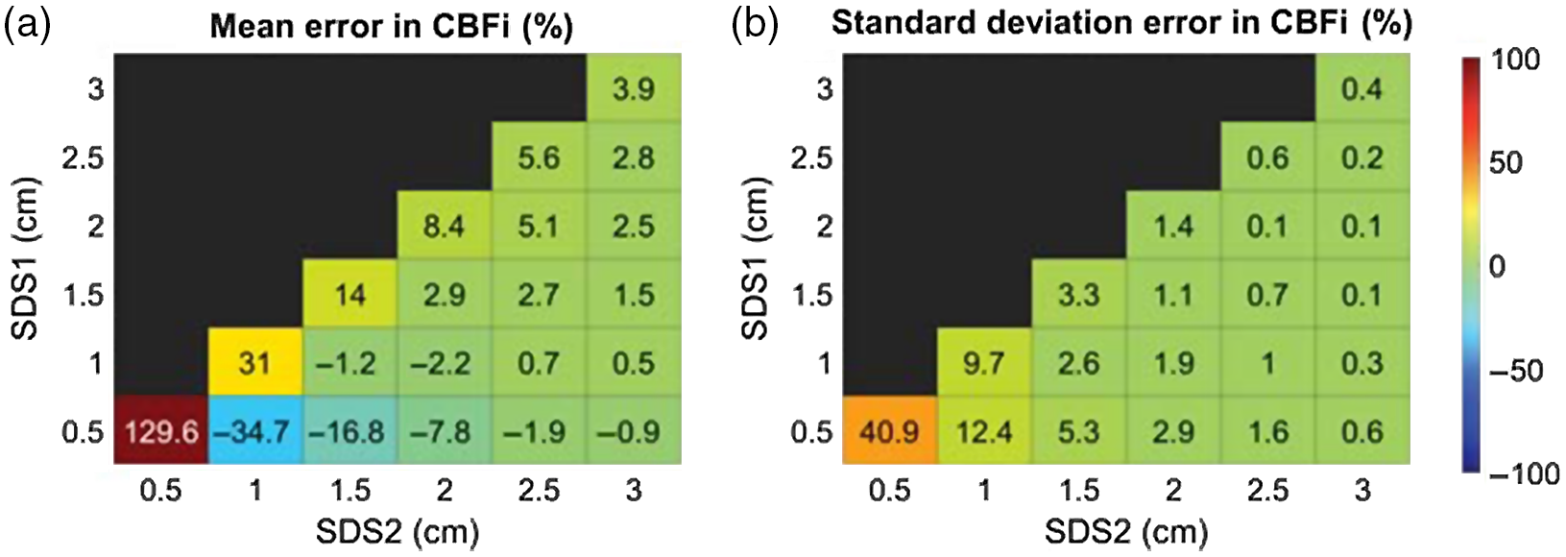
Error of estimated CBFi when using MC simulated data at different SDS. Each box represents the (a) mean and (b) standard deviation in the error of CBFi across 72 samples of varying scalp and cerebral blood flow indices (6 SBFi × 12 CBFi) for the SDS combination given the row and column.

**Fig. 6:**
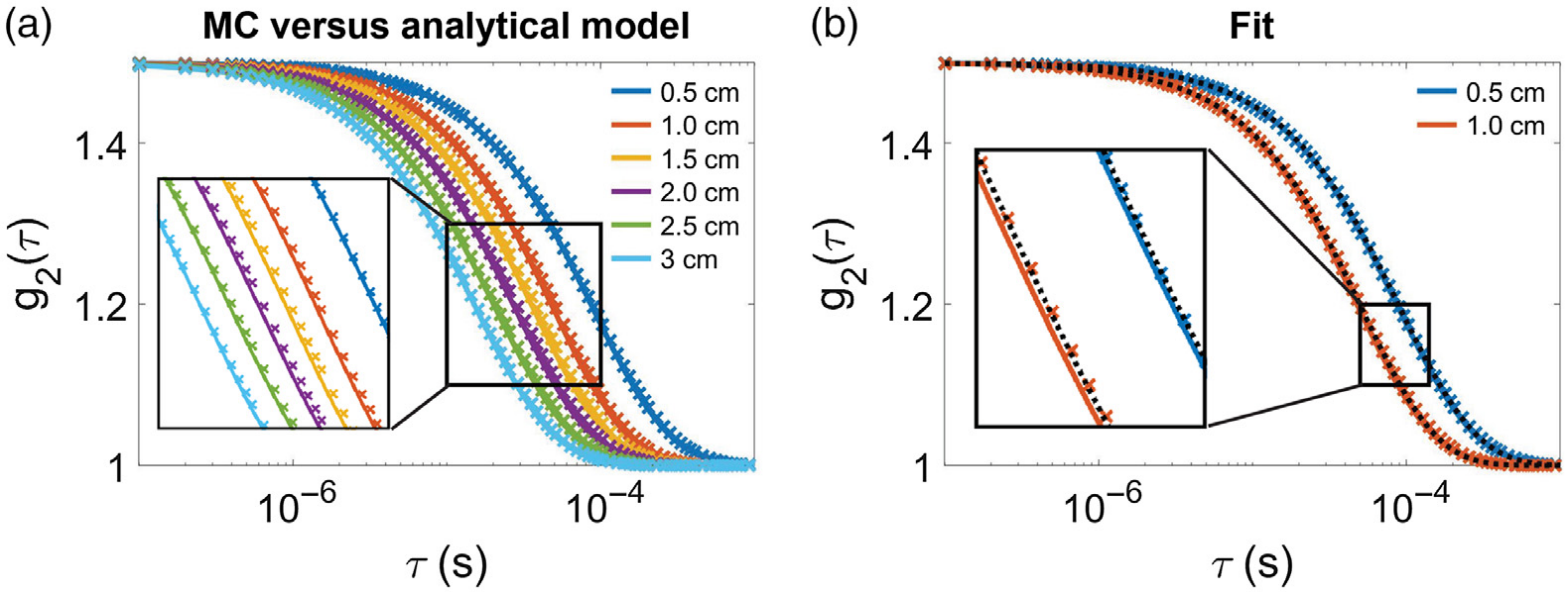
(a) Representative $g_{2}(\tau )$ estimated with MC on a three-layer slab (represented with colored cross markers) along with the corresponding analytical solution for this geometry and optical/dynamic property combination (solid lines). (b) The MC simulated data from (a) at 0.5 and 1.0 cm (shown in × markers), along with the corresponding analytical solution for this geometry and optical/dynamic property combination (solid line), and the best fit of the MC data to the three-layer analytical model (black dashed line). In this dataset, SBFi and CBFi were set to $1.7\times 10^{-8}$ and $5.2\times 10^{-8}\;{\mathrm{cm}}^{2}/s$, respectively, while the best fit estimates of SBFi and CBFi were $1.7\times 10^{-8}$ and $2.2\times 10^{-8}\;{\mathrm{cm}}^{2}/s$, reflecting a 0% error in SBFi but a −58% error in CBFi. In both subplots, the inset displays a magnified view of the deviation between MC simulated data and the analytical solution.

**Fig. 7:**
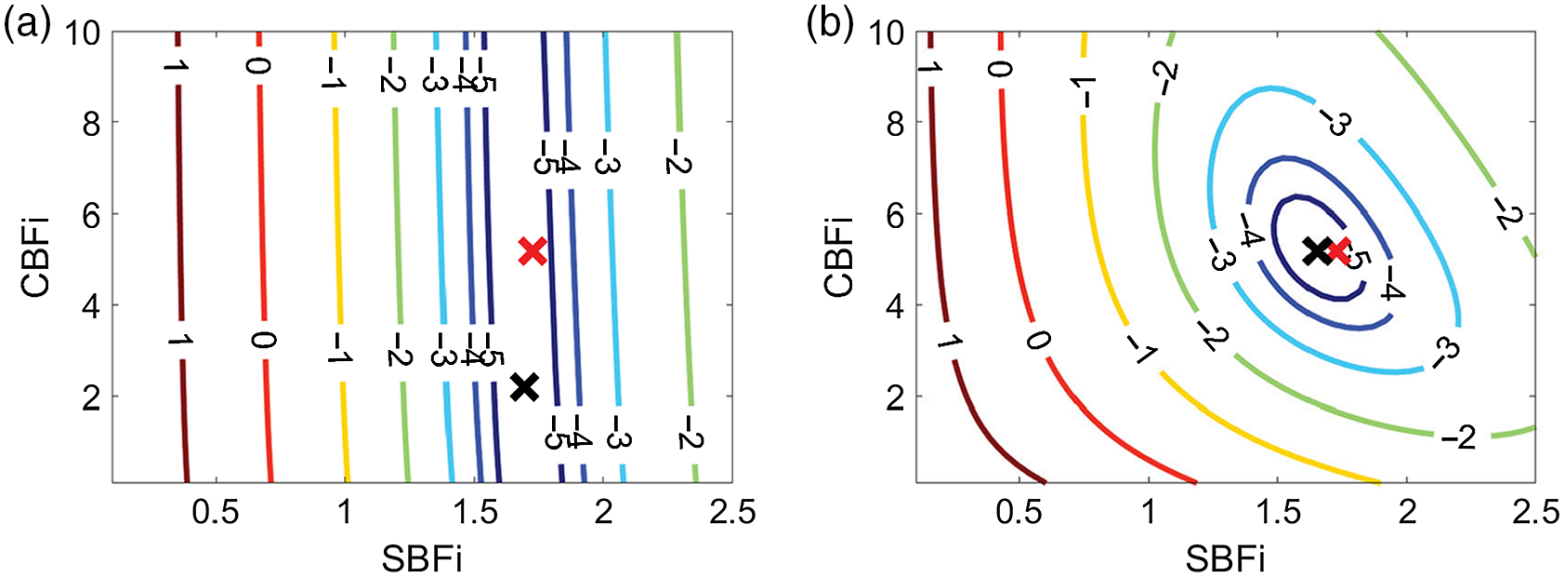
Representative contour plots of the log of the cost function, $\chi^{2}$ when using three-layer analytical model to fit the MC simulated data with different combinations of two SDSs. Here, $\chi^{2}$ is plotted as a function of SBFi and CBFi (in units of $1\times 10^{-8}\;{\mathrm{cm}}^{2}/s$). For clarity, contour lines for which $\log(\chi^{2})<-5$ are not plotted. In each subplot, the true CBFi and SBFi values are denoted by a red cross sign, and the fitted values are denoted by a black cross sign. (a) SDSs combinations of 0.5 and 1 cm, (b) SDS combinations of 1 and 3 cm.

We further evaluated the accuracy of the three-layer estimations of CBFi using MC simulations on a three-layer slab medium (Fig. 5). In general, the MC results mirror the results of the three-layer inverse model (Figs. 2 and 4) when using two SDS >1  cm. However, there are distinguishable errors in CBFi when using a single SDS at 0.5 or 1 cm, or when using two SDS at 0.5 and 1 cm or 0.5 and 1.5 cm. Comparing simulated $g_{2}(\tau )$ from the analytical model with those from MC, we found that the MC simulated curves were slightly right shifted at all SDS [Fig. 6(a)]. The influence of this right shift on the accuracy of CBFi estimation is most prominent for small SDS due to the shape of the cost function [Fig. 7(a)]. For small SDS, the minimum of the cost for a given SBFi falls along a wide range of CBFi, such that small deviations from the true $g_{2}(\tau )$ curve can yield large errors in CBFi. The influence of this right shift is less prominent for SDS combinations like 1 and 3 cm, wherein the minimal cost is confined to a tight area around the true value [Fig. 7(b)].

We note that the error in CBFi estimated from MC simulated data is much less than the error in CBFi estimated from the noise-added three-layer analytical model. This result suggests that measurement noise is the dominant influence in the accuracy of the three-layer analytical model. Thus, for the remainder of the results, we use data simulated with the three-layer analytical model with noise added to investigate the sensitivity of CBFi to inaccuracies of the assumed model parameters.

### 3.3 Sensitivity to Model Parameters Depends on Source–Detector Separation

**Fig. 8:**
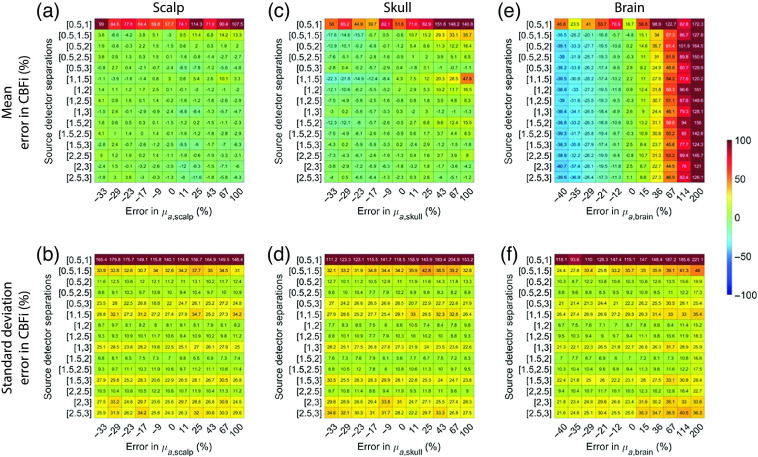
Error in estimated CBFi as a function of error in assumed values in the absorption coefficient, $\mu_{a}$, of each layer. Each row reflects the SDS combination used to fit for CBFi, and each column reflects the percent error in the assumed value of $\mu_{a}$ for (a) and (b) the scalp, (c) and (d) skull, or (e) and (f) brain layers. Results are presented as mean (a), (c), and (e) and standard deviation (b), (d), and (f) of the error in the estimated CBFi across all 72 combinations of simulated SBFi and CBFi. Data were simulated with noise added (detected intensity of 20 kHz at 2.5 cm, integration time of 10 s).

**Fig. 9:**
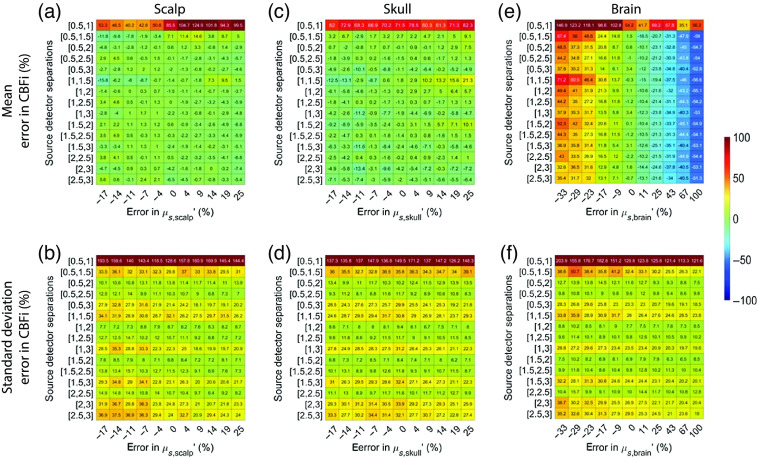
Error in estimated CBFi as a function of error in assumed values in the reduced scattering coefficient, $\mu_{s}^{\prime }$, of each layer. Each row reflects the SDS combination used to fit for CBFi, and each column reflects the percent error in the assumed value of $\mu_{s}^{\prime }$ for (a) and (b) the scalp, (c) and (d) skull, or (e) and (f) brain layers. Results are presented as mean (a), (c), and (e) and standard deviation (b), (d), and (f) of the error in the estimated CBFi across all 72 combinations of simulated SBFi and CBFi. Data were simulated with noise added (detected intensity of 20 kHz at 2.5 cm, integration time of 10 s).

In total, regardless of SDS, the estimation of CBFi is most sensitive to inaccuracies in brain optical properties compared with inaccuracies in scalp and skull optical properties (Figs. 8 and 9). The magnitude of this sensitivity is relatively independent of the choice of SDS, with the exception of the 0.5 and 1 cm combination, which had large errors in CBFi, and the combinations containing 3 cm wherein the standard deviation of error across all samples tested was slightly larger. The trends suggested that under-/over-estimations of the absorption coefficient of the brain layer can cause under-/over-estimations of CBFi, respectively, while under-/over-estimations of the reduced scattering coefficient of the brain layer can cause over-/under-estimations of CBFi, respectively.

**Fig. 10:**
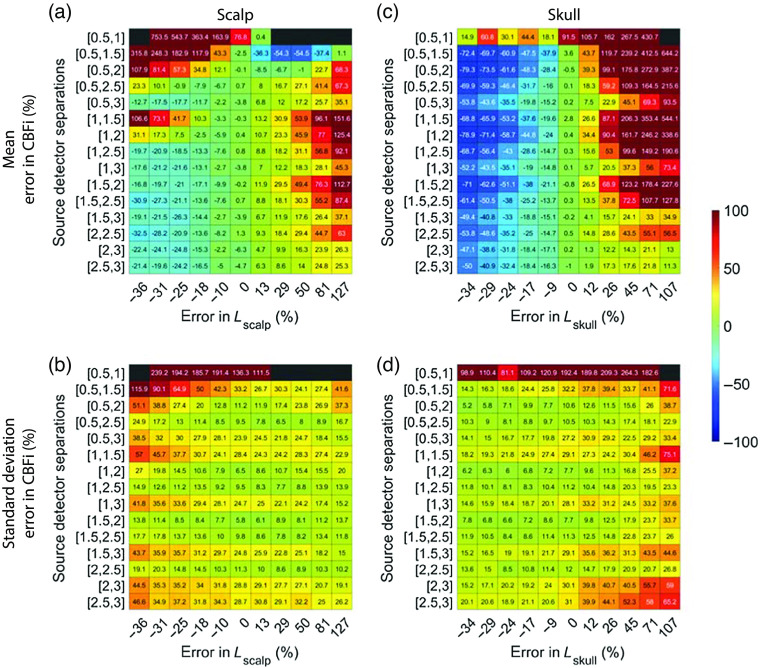
Error in estimated CBFi as a function of error in assumed values in the thickness, L, of each layer. Each row reflects the SDS combination used to fit for CBFi, and each column reflects the percent error in the assumed value of L for (a) and (b) the scalp or (c) and (d) skull layers. Results are presented as (a) and (c) mean and (b) and (d) standard deviation of the error in the estimated CBFi across all 72 combinations of simulated SBFi and CBFi. Data were simulated with noise added (detected intensity of 20 kHz at 2.5 cm, integration time of 10 s).

Additionally, inaccuracies in both scalp and skull thickness can lead to nonnegligible errors in CBFi (Fig. 10). On average, under-/over-estimation of both scalp and skull thickness caused under-/over-estimation of CBFi (Fig. 10). However, in contrast to optical properties, the magnitude of these errors is highly dependent on the choice of SDS. On average, source–detector combinations that include 3 cm were least sensitive to errors in both scalp and skull thickness, although the standard deviation across all samples tested was large for these combinations of SDS. Combinations of 0.5 and 1 cm and 0.5 and 1.5 cm led to the greatest sensitivity to errors in scalp and skull thickness.

## 4 Discussion

In this study, we determined the influence of the SDS on the accuracy of the estimation of cerebral blood flow using the three-layer model across a wide range of samples with varying optical properties, thicknesses, and varying dynamics. As expected, our results indicate that the choice of SDS heavily influenced both the uniqueness of the CBFi solution, and the accuracy of its estimation, as well as the sensitivity of the estimation to model parameters. Thus, when using this model, the choice of SDS is an important consideration.

**Fig. 11:**
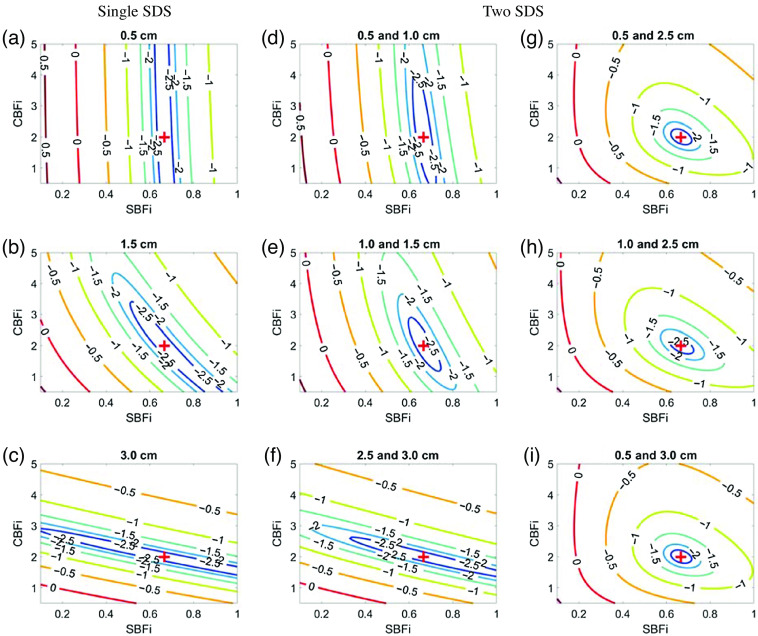
Representative contour plots of the log of the cost function, $\chi^{2}$ when using (a)–(c) a single SDS or (d)–(i) a combination of two SDSs to fit the clean data. Here, $\chi^{2}$ is plotted as a function of SBFi and CBFi (in units of $1\times 10^{-8}\;{\mathrm{cm}}^{2}/s$). For clarity, contour lines for which $\log(\chi^{2})<-2.5$ are not plotted. In each subplot, the true CBFi and SBFi values are denoted by a red plus sign. (d)–(f) a pair of closely spaced detectors and (g)–(i) a larger gap between detectors.

When using a single SDS to estimate CBFi with the three-layer model, we found that there are some samples for SDS≥1.5  cm without a unique solution (as indicated by the nonzero error in Fig. 2). The combination of fitting for two parameters (CBFi and SBFi) as well as the shape of the cost map when using a single SDS (Fig. 11) leaves an illposed problem for which a unique solution does not exist. Further, our MC results reveal that, in the case of a single small (≤1  cm) SDS, slight deviations in the measured $g_{2}(\tau )$ data from the analytical model can lead to significant errors in CBFi (Fig. 5). Thus, the use of a single SDS is not recommended with the three-layer model due to nonuniqueness and sensitivity to noise.

In contrast to a single SDS, two SDS generally provided a more accurate estimation of CBFi when tested with noisy data and in MC simulations. However, if both SDS <2  cm, the estimation of CBFi is highly sensitive to noise (Fig. 4). This sensitivity can be explained by the shape of the cost map [Figs. 11(d) and 11(e)], which is tightly constrained along the direction of SBFi rather than CBFi because detected photons at these small separations mainly interact with superficial layers. On the other end of the spectrum, when using two large SDSs (both >2  cm), although the cost map is tightly constrained along the direction of CBFi rather than SBFi [Fig. 11(f)], the increase in noise due to reduced intensity leads to errors in CBFi estimation (Fig. 4). Thus, our results suggest that the optimal combination of two SDS should contain one SDS <2  cm and one at 2.5 cm. These SDS combinations provide a tightly bound cost map (Figs. 7 and 11) and were associated with the lowest errors in CBFi estimation (Figs. 4 and 5). Moreover, these combinations also minimized the influence of errors in assumed optical properties and layer thickness on the estimation of CBFi compared with other SDS combinations (Figs. 8–10). However, while minimized, the error due to inaccurate model parameters can still be appreciable, as previously discussed.^16^ In sum, for the intensities and SDS combinations tested, we found that utilizing two SDS, in which one SDS is <2  cm and the other SDS is at 2.5 cm, provides the optimal combination to balance the depth penetration with the SNR to minimize the error in CBFi. We note that, by adding more detectors to the experimental setup, e.g., a 16 channel DCS system, the optimal location of these two SDS will likely shift to larger values.

**Fig. 12:**
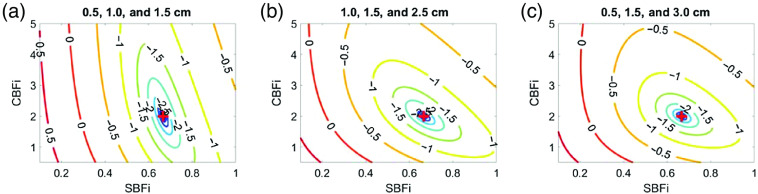
Representative contour plots of the log of the cost function, $\chi^{2}$ when using a combination of three SDSs to fit the data. Here, $\chi^{2}$ is plotted as a function of SBFi and CBFi (in units of $1\times 10^{-8}\;{\mathrm{cm}}^{2}/s$). For clarity, contour lines for which $\log(\chi^{2})<-2.5$ are not plotted. In each subplot, the true CBFi and SBFi values are denoted by a red plus sign. (a) a pair of closely spaced detectors at small SDS, (b) a pair closely spaced at larger SDS, and (c) a larger gap between detectors.

This study has several limitations. First, we only considered either a single SDS or a pair of SDS. We found that adding a third SDS did not appreciably change the contours of the cost map compared with that of two SDS (Fig. 11 versus Fig. 12). Second, in our noise-added simulations, we simulated a DCS system with eight total detectors. We placed these detectors at either a single SDS or at two SDS wherein the second SDS contains a bundle of seven detectors that are averaged together to mimic commonly observed experimental conditions. Considering the significant expense of adding single photon counting module detectors to a traditional continuous-wave DCS system, our results suggest that two SDS maximize the cost-to-accuracy ratio. As the photon efficiency and bin width of novel single photon avalanche diode arrays that contain hundreds of detectors for DCS detection continue to improve, these detectors may enable collection at many SDS, which could greatly improve the accuracy of the three-layer inverse problem. Third, we limited our investigation to a separation range between 0.5 and 3 cm; we chose this range because of low detected intensities at SDS >3.0  cm^2^^,^^25^ and possible break down of correlation diffusion theory at SDS <0.5  cm.^26^^,^^27^ Fourth, because we were interested in evaluating the performance of a three-layer analytical model at different SDSs, we only validated the accuracy with a three-slab MC simulation. However, the slab model grossly simplifies the complex structure of the human head. Neither curvature, CSF, nor heterogeneity within each layer was considered. Fifth, we fixed the coherence factor (β) at 0.5 in this investigation. However, in real experiments wherein β is not known, error in estimating the coherence factor may further increase the error in estimated CBFi. Finally, because the magnitude of the mean/stdev errors reported in Fig. 4 are unique to the simulated detected intensity (20 kHz at 2.5 cm for this study), the results should be used as guidance of the general trends in the estimation of CBFi with the three-layer model as a function of SDS, specific to the case of continuous-wave DCS. Future work should translate these findings to simulations that employ more realistic head geometries, e.g., by utilizing segmented anatomical images from magnetic resonance imaging or computed tomography (CT), as well as validate this approach *in vivo* against other gold standard perfusion modalities. Further, although we qualitatively show that the cost maps generated from two versus three SDS look similar (Fig. 12), it is worth investigating whether combinations of more SDS could improve CBFi estimation with the addition of more detectors.^28^^,^^29^

## 5 Conclusion

We demonstrated that the choice of SDS is critical for minimizing the estimate error of cerebral blood flow when using the three-layer analytical model to analyze DCS data. Two SDS were required to ensure a unique solution for CBFi. We found combinations of 0.5/1.0/1.5 cm and 2.5 cm provided the optimal choice to balance the depth penetration with the SNR to minimize the error in CBFi across a wide range of samples with varying optical properties, thicknesses, and dynamics.

## 6 Appendix: Sensitivity to Model Parameters in Clean Signal

**Fig. 13:**
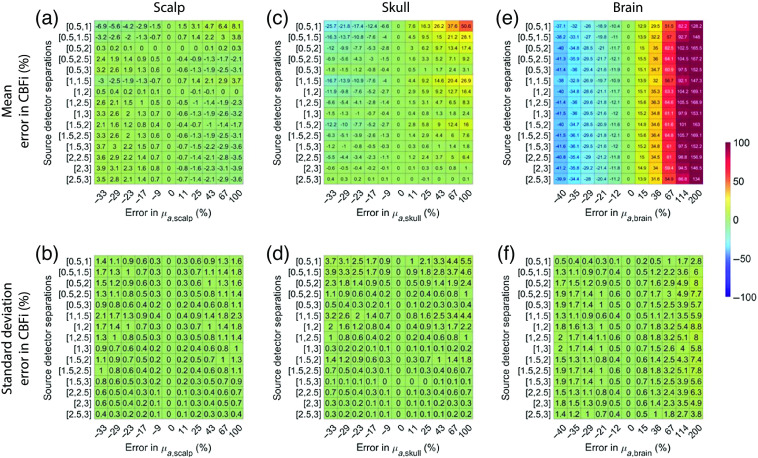
Error in estimated CBFi as a function of error in assumed values in the absorption coefficient, $\mu_{a}$, of each layer. Each row reflects the SDS combination used to fit for CBFi, and each column reflects the percent error in the assumed value of $\mu_{a}$ for (a) and (b) the scalp, (c) and (d) skull, or (e) and (f) brain layers. Results are presented as mean (a), (c), and (e) and standard deviation (b), (d), and (f) of the error in the estimated CBFi across all 72 combinations of simulated SBFi and CBFi. Data were simulated without noise.

**Fig. 14:**
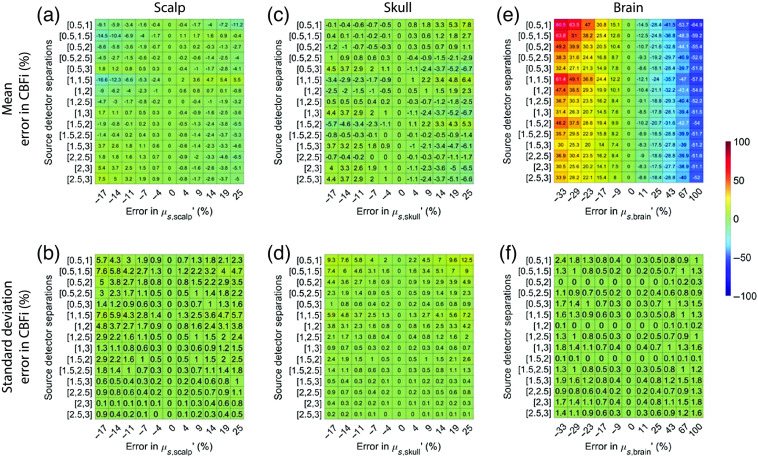
Error in estimated CBFi as a function of error in assumed values in the reduced scattering coefficient, $\mu_{s}^{\prime }$, of each layer. Each row reflects the SDS combination used to fit for CBFi, and each column reflects the percent error in the assumed value of $\mu_{s}^{\prime }$ for (a) and (b) the scalp, (c) and (d) skull, or (e) and (f) brain layers. Results are presented as mean (a), (c), and (e) and standard deviation (b), (d), and (f) of the error in the estimated CBFi across all 72 combinations of simulated SBFi and CBFi. Data were simulated without noise.

**Fig. 15:**
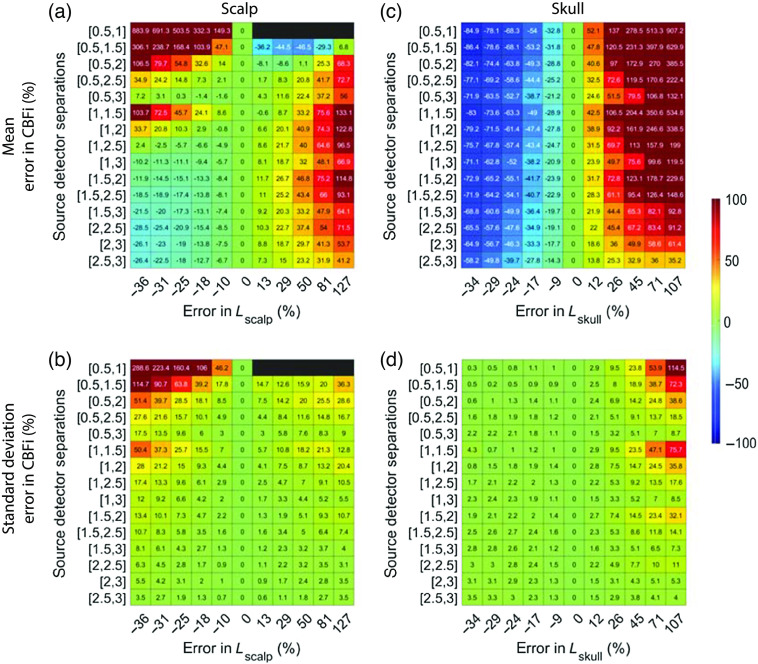
Error in estimated CBFi as a function of error in assumed values in the thickness, L, of each layer. Each row reflects the SDS combination used to fit for CBFi, and each column reflects the percent error in the assumed value of L for (a) and (b) the scalp or (c) and (d) skull layers. Results are presented as mean (a) and (c) and standard deviation (b) and (d) of the error in the estimated CBFi across all 72 combinations of simulated SBFi and CBFi. Data were simulated without noise.

For reference, we present the results from Figs. 8–10, which were obtained with noise added data, using clean data in Figs. 13–15. In general, the distribution of mean errors in estimated CBFi across different combinations of SDSs obtained from a clean signal are similar to the results from the noise-added signal. However, the standard deviation of the error in estimated CBFi for the clean data is relatively smaller when using combinations of two large SDSs because the higher brain sensitivity is not clouded by the influence of increased noise.

## Data Availability

Data underlying the results presented in this paper are not publicly available at this time but may be obtained from the authors upon reasonable request.
